# Islet Autoantibodies in Adult With Pituitary and Ovarian Autoimmunity: Implications for Type 1 Diabetes Screening

**DOI:** 10.1111/dom.70776

**Published:** 2026-04-20

**Authors:** Giuseppe Bellastella, Michela di Nuzzo, Miriam Longo, Paolo Cirillo, Antonietta Maio, Nicole Di Martino, Silvia Angelino, Paola Caruso, Lorenzo Scappaticcio, Maria Teresa Vietri, Maria Ida Maiorino, Katherine Esposito

**Affiliations:** ^1^ Unit of Endocrinology and Metabolic Diseases AOU University of Campania “Luigi Vanvitelli” Naples Italy; ^2^ Department of Advanced Medical and Surgical Sciences University of Campania “Luigi Vanvitelli” Naples Italy; ^3^ Department of Life Science, Health, and Health Professions Link Campus University Rome Italy; ^4^ Department of Precision Medicine University of Campania “Luigi Vanvitelli” Naples Italy

**Keywords:** autoimmunity, islets, pancreatic autoantibodies, type 1 diabetes

## Abstract

**Purpose:**

Type 1 diabetes is a chronic autoimmune disease characterised by progressive pancreatic β‐cell destruction and the early appearance of islet autoantibodies (islet‐AAb) during the pre‐symptomatic phases. Given the growing interest in screening and prevention of type 1 diabetes, this study aimed to assess the prevalence of pancreatic autoimmunity in adults with other organ‐specific autoantibodies in order to identify potential target populations for screening programs.

**Methods:**

Adults previously tested for autoantibodies against specific endocrine glands and/or gastric parietal cells by indirect immunofluorescence were divided into two groups (Autoimmunity group and Control group), according to the presence of at least one organ‐specific autoantibody. Stored serum samples from all participants were re‐evaluated for islet‐AAb and comparisons were performed between groups and, within the Autoimmunity group (Group A), across different patterns of organ‐specific autoimmunity.

**Results:**

Positivity for at least one islet‐AAb was significantly higher in Group A than in Control Group (40.3% vs. 21.0%; *p* = 0.007). Double and triple islet‐AAb positivity were less common overall, but occurred more frequently in Group A. Moreover, in Group A, a significantly higher prevalence of multiple positivity (≥ 2 islet‐AAb) was observed in subjects with ovarian autoimmunity and in those with combined pituitary and ovarian autoimmunity than in those with other endocrine autoimmune involvement.

**Conclusion:**

Adults with specific autoimmunity disorders, particularly those affecting ovarian and/or pituitary glands, have a higher prevalence of pancreatic autoimmunity. These subjects may benefit from targeted screening to identify the pre‐symptomatic stage of T1D and potentially delay progression to clinical disease.

## 1 Introduction

Type 1 diabetes (T1D) is an organ‐specific autoimmune disease whose incidence has been rapidly increasing in many countries [1]. It is characterised by insulin deficiency resulting from progressive pancreatic *β*‐cell destruction and the knowledge of its pathogenesis has markedly expanded over recent decades [2]. T1D develops through distinct stages, and individuals with a single islet autoantibody (islet‐AAb) are considered at increased risk and require regular monitoring. Islet‐AAb include autoantibodies against glutamic acid decarboxylase (anti‐GAD), zinc transporter 8 (anti‐ZNT‐8), tyrosine phosphatase‐like protein (anti‐IA2) and anti‐insulin (IAA) [3]. The first stage is a pre‐symptomatic phase, defined by the serological positivity for two or more islet‐AAb, in the absence of dysglycaemia; stage 2 is characterised by glycaemic alterations not yet fulfilling diagnostic criteria for diabetes. Stage 3 corresponds to clinical diabetes with overt symptoms and diagnostic dysglicaemia [3]. Initial manifestations often include weight loss, polyuria and polydipsia and diagnosis of T1D may be delayed until hospital admission for diabetic ketoacidosis (DKA), a life‐threatening condition that adversely affects survival, morbidity and quality of life for patients and their families [4]. Timely identification of individuals at risk or in the early pre‐symptomatic stages through screening programs allows structured counselling, prevention of severe acute presentations and where available, the use of emerging therapies that can delay disease progression [3]. Teplizumab, a humanised anti‐CD3 monoclonal antibody, has been shown to delay onset of clinical T1D by a median of 24 months and it is now approved or available for compassionate use in several countries for individuals aged 8 years and older with stage 2 of disease [5]. Currently, islet‐AAb are the most reliable serum markers of β‐cell autoimmunity for screening, although the prognostic value of their number, titre, and specificity differs across age [3]. According to the American Diabetes Association (ADA), autoantibody‐based screening is recommended for individuals with a family history of T1D or otherwise known elevated genetic risk [6]. Most existing screening programs have primarily targeted high‐risk HLA genotypes and were subsequently extended to the general paediatric population [7]. T1D is traditionally regarded as a childhood‐onset disease, but it can occur at any age and further investigation is needed in adults with T1D. Adult‐onset autoimmune diabetes is clinically and pathogenically heterogeneous, is often misclassified with type 2 diabetes and shows variable rates of beta‐cell loss, insulin requirement and risk of ketoacidosis, making prompt and accurate diagnosis crucial for proper management [8]. The presence of concomitant autoimmune disorders may help identify adults with autoimmune diabetes, as T1D often coexists with other autoimmune disorders affecting endocrine glands, such as autoimmune polyglandular syndromes (APS) [9], or non‐endocrine tissues, including coeliac disease, atrophic gastritis, multiple sclerosis and psoriasis [10]. APS type 1 is classically defined by the presence of at least two of the following conditions: chronic mucocutaneous candidiasis, hypoparathyroidism and Addison's disease. APS type 2 is characterised by the association of Addison's disease with autoimmune thyroid disease and/or T1D. APS type 3 refers to autoimmune thyroid disease occurring in combination with one or more other autoimmune disorders, in the absence of Addison's disease. Finally, APS type 4 includes combinations of autoimmune endocrine and non‐endocrine diseases that do not fulfil the criteria for the other APS types [9]. While people with T1D are known to be at increased risk of other autoimmune diseases [10, 11], the reverse association of developing T1D in the presence of other organ‐specific autoimmunity remains less explored and available data largely derive from paediatric cohorts [12, 13]. In light of the growing attention in T1D screening and the recognised need to strengthen the evidence base in adult‐onset disease, the present study aimed to assess the prevalence of islet‐AAb in a cohort of adults with at least one organ‐specific autoimmune condition and across the different types of autoimmunity, in order to identify potential target populations for future screening programs.

## 2 Materials and Methods

### 2.1 Study Population

A total of 210 subjects with overt or suspected autoimmune diseases, previously evaluated for at least one antibody against endocrine glands (pituitary gland, hypothalamus, adrenal cortex, ovary) or gastric parietal cells (GPCs), were identified from the database of the Immunoendocrinology laboratory at the University Hospital Luigi Vanvitelli, Naples, Italy. Exclusion criteria at the time of blood sampling for organ‐specific autoimmunity were: (1) age < 18 years, (2) diagnosis of type 1 or type 2 diabetes, (3) family history of type 1 diabetes, (4) current acute illness or acute illness within the previous 6 months, (5) pregnancy or breastfeeding, (6) current or recent (within 6 months) immunosuppressive therapy, (7) active malignancy, and (8) lack of an adequate serum sample. Subjects receiving current or recent immunosuppressive therapy were excluded to minimise potential interference with autoantibody detection and to avoid misclassification of pancreatic autoimmunity. Participants were divided into two groups: an Autoimmunity group (Group A) including 134 subjects presenting one or more organ‐specific autoimmunity against endocrine glands and/or GPCs and a control group (CTRLs) consisting of 76 individuals with no evidence of autoimmunity and no documented history of autoimmune diseases, although occult autoimmune conditions could not be completely excluded. The study was approved by the Medical Ethics Committee of University of Campania ‘Luigi Vanvitelli’ (Prot.0032232/i of 28/11/2025) and conducted in accordance with the ethical standards of the institutional and/or national research committee and with the 1964 Helsinki declaration and its later amendments or comparable ethical standards. Written informed consent was obtained from all participants.

### 2.2 Data

For each participant, data retrieved from medical records included sex, age and the presence or absence of diabetes, coeliac disease and autoimmune thyroid disease. Serum samples from both groups were stored under controlled conditions in our laboratory and subsequently analysed for the presence of the following islet‐AAb: anti‐GAD, anti‐IA2, anti‐Znt8 and anti‐insulin. In Group A, the prevalence of islet‐AAb was further assessed within each category of organ‐specific autoimmunity, considered either alone or in combination.

### 2.3 Autoimmunity Assessment

Organ‐specific autoantibodies had been assessed by indirect immunofluorescence, as previously described [14, 15, 16, 17]. Unfixed cryostat sections of endocrine glands including pituitary, hypothalamus, adrenal cortex, ovary and stomach from young healthy baboons were initially incubated with participants' sera, followed by fluorescein isothiocyanate‐conjugated goat anti‐human immunoglobulin (Li StarFish, Cernusco sul Naviglio, Milano, Italy) to detect the presence of organ‐specific autoantibodies. On this basis, antipituitary (APA), antihypothalamus (AHA), anti–adrenal cortex (ACA), anti‐ovarian (AOA) and anti–gastric parietal cell (GPCs) antibodies were identified. To increase the specificity of the method, samples were considered positive at titre ≥ 1:8.

Pancreatic autoimmunity was evaluated by measuring serum levels of anti‐GAD (MEDIZYM ANTI GAD Cod. Costr. 3802 PANTEC SRL), anti‐IA2 (MEDIZYM ANTI IA2 Cod. Costr. 3803 PANTEC SRL), anti‐Znt8 (ELISARSR ZNT8AB Cod. Costr. ZNT8/96 PANTEC SRL) and anti‐insulin (MEDIZYM ANTI IAA‐Cod. Costr. 3806 PANTEC SRL) using solid‐phase sandwich enzyme‐linked immunosorbent assay (ELISA). Samples were processed on an automated analyser (Triturus, PANTEC, Serial Number 0530001900) according to the manufacturer's instructions. Positivity thresholds were: GADA ≥ 5.0 UI/mL, Znt8A ≥ 15.0 UI/mL, IA2A ≥ 10 UI/mL and IAA ≥ 2.4 UI/mL. Reported assay sensitivities and specificities were 98.4% and 95.3% for anti‐GAD, 94.2% and 93.1% for anti‐IA2, 77% and 94% for IAA, 72% and 99% for anti‐Znt‐8 AAb.

### 2.4 Statistical Analysis

Descriptive statistics were used to characterise the study population. Continuous variables were presented as mean and standard deviation (SD) and categorical variables as frequencies and percentages. Differences between groups were assessed using two‐sided Student's *t*‐test for continuous variables and *χ*2‐test for dichotomous variables. Statistical significance was accepted at *p* < 0.05.

To our knowledge, no studies have been performed to evaluate the prevalence of pancreatic autoimmunity in subjects with the different organ‐specific autoimmunities that we analysed. Published data are limited to subjects with autoimmune thyroid disease. Based on this evidence, we estimated an expected prevalence (*p*) of pancreatic autoimmunity in our study group (Group A) of approximately 10%. The statistical precision of this estimate was then calculated using the formula for the margin of error (*E*): *E* = *Z* × √(*p*(1 − *p*)/*n*), where *Z* = 1.96 (for a 95% confidence level), *p* = 0.10 (the expected prevalence) and *n* = 134 (the sample size for Group A). This gave us an estimated margin of error of: *E* = 1.96 × √(0.10 × (1–0.10)/134) ≅ 0.051.

Therefore, our sample size of 134 subjects with organ‐specific autoimmunity was sufficient to estimate the prevalence of pancreatic autoimmunity with a 95% confidence level and a margin of error (precision) of approximately 5.1%.

## 3 Results

The whole study population had a median age of 39 years (25.0–49.0); 66.6% (*n* = 140) were female and 29.5% (*n* = 62) were obese. Pancreatic autoimmunity, defined as serum positivity for at least one islet‐AAb, was detected in 33.0% of participants (*n* = 70). Group A and CTRLs did not differ significantly in terms of age (39.0 ± 16.4 vs. 36.2 ± 15.1 years, *p* = 0.239) or sex distribution. Obesity was more prevalent in Group A than in CTRLs, although not significantly. Positivity for at least one islet‐AAb was significantly more frequent in Group A than in CTRLs (*n* (%), 54 (40.3) vs. 16 (21.0), *p* = 0.007); double and triple islet‐AAb positivity were less common overall but occurred more frequently in Group A (Table 1).

**TABLE 1:** Clinical characteristics of partecipants in the study.

Variables	Group A (*n* = 134)	CTRLs (*n* = 76)	*p*
Age, years	39.0 ± 16.4	36.2 ± 15.1	0.239
Gender, *n* (%)			0.204
Female	94 (70.1)	46 (60.5)	
Male	40 (29.9)	30 (39.5)	
Obesity, *n* (%)	46 (34.3)	16 (21.0)	0.062
Islet‐AAb *n* (%)			
One islet AAb	54 (40.3)	16 (21.0)	0.007
Two islet AAb	12 (8.9)	4 (5.2)	0.485
Three islet AAb	4 (3.0)	2 (2.6)	0.777

Abbreviation: Islet‐AAB, islet autoantibodies.

Table 2 summarises the prevalence of each islet‐AAb in the two study groups, considering both single and multiple positivity (≥ 2 islet‐AAb). In group A, anti‐IA2 AAb was the most frequent autoantibody and its prevalence was significantly higher than in CTRLs (34.3% vs. 5.3%, *p* < 0.001); no significant differences between groups were observed for anti‐GAD, anti‐IAA, or anti‐ZnT8A. Among participants with only one positive islet‐AAb, anti‐IA2 predominated in group A, anti‐GAD was more frequent in CTRLs and no subject in CTRLs was positive for IAA alone. Consequently, patterns of multiple islet‐AAb positivity differed between groups, with combined anti‐GAD and anti‐IA2 positivity being more frequent in group A than in CTRLs (*p* = 0.07).

**TABLE 2:** Prevalences of islet‐AAb in study groups.

Variables	Group A (*n* = 134)	CTRLs (*n* = 76)	*p*
Islet AAb, *n* (%)			
Anti‐GAD	16 (11.9)	12 (15.8)	0.564
IAA	6 (4.5)	0 (0)	0.150
Anti‐IA2	46 (34.3)	4 (5.3)	< 0.001
Anti‐Znt8	2 (1.5)	6 (7.9)	0.741
One islet AAb, *n* (%)			
Anti‐GAD	4 (3)	8 (10.5)	0.056
IAA	4 (3)	0 (0)	0.329
Anti‐IA2	34 (25)	2 (2.6)	< 0.001
Anti‐Znt8	0 (0)	2 (2.6)	0.251
Two islet AAb (*n*)			
Anti‐GAD + IA2	8	0	0.07
Anti‐GAD + ZnT8	0	2	0.251
Three islet AAb (*n*)			
Anti‐GAD + IAA + IA2	2	0	0.741
Anti‐GAD + IAA2 + Znt8	2	2	0.956

Abbreviations: anti‐GAD, anti‐glutamic acid decarboxylase; anti‐IA2, anti‐tyrosine phosphatase‐like protein; anti‐ZNT8, anti‐zinc transporter 8; CTRLs, control group; Group A, autoimmunity group; IAA, antobodies anti‐insulin; Islet‐AAB, islet autoantibodies.

To better characterise the two groups, islet‐AAb titers were categorised as low, medium or high, corresponding to values, respectively, below the 25th percentile, at the 50th percentile and above the 75th percentile of the overall study population (Table 3). In Group A, the mean titre was high for anti‐GAD, anti‐Znt8 and anti‐IA2 and medium‐high for IAA, whereas in CTRLs, the mean titre was low for anti‐GAD and anti‐IA2 and medium‐low for anti‐Znt8. When restricting the analysis to individuals with multiple islet‐AAb positivity, these patterns were confirmed in Group A, while in CTRLs mean titers were medium‐high for anti‐GAD, medium‐low for anti‐Znt8 and low for anti‐IA2 (Table 4).

**TABLE 3:** Titre of islet‐AAb in study groups.

Islet‐AAb	Cut‐off, UI/ml	Medium titre, UI/ml	Low‐titre, UI/ml	High‐titre, UI/ml	Group A (mean titre ± SD)	CTRLs (mean titre ± SD)
Anti‐GAD	5	11.95	8.3	15.0	20.3 ± 18.0	10.6 ± 6.3
Anti‐Znt8	15	19.8	18.2	22.9	26.8 ± 2.1	19.0 ± 1.5
Anti‐IA2	8	15.7	12.1	20.7	24.1 ± 21.5	8.5 ± 0.4
IAA	2.4	4.0	3.6	5.2	4.2 ± 0.7	—

Abbreviations: anti‐GAD, anti‐glutamic acid decarboxylase; anti‐IA2, anti‐tyrosine phosphatase‐like protein; anti‐ZNT8, anti‐zinc transporter 8; CTRLs, control group; Group A, autoimmunity group; IAA, antobodies anti‐insulin; Islet‐AAB, islet autoantibodies; SD, standard deviation.

**TABLE 4:** Titre of islet‐AAb in partecipants with ≥ 2 islet‐AAb.

Islet‐AAb	Cut‐off, UI/ml	Medium Titre, UI/ml	Low‐titre, UI/ml	High‐titre, UI/ml	Group A (mean titre ± SD)	CTRLs (mean titre ± SD)
Anti‐GAD	5	11.95	8.3	15.0	22.5 ± 20.4	13.8 ± 9.0
Anti‐Znt8	15	19.8	18.2	22.9	26.8 ± 2.1	19.8 ± 0.9
Anti‐IA2	8	15.7	12.1	20.7	35.8 ± 17.9	8.5 ± 0.4
IAA	2.4	4.0	3.6	5.2	4.0 ± 0	—

Abbreviations: anti‐GAD, anti‐glutamic acid decarboxylase; anti‐IA2, anti‐tyrosine phosphatase‐like protein; anti‐ZNT8, anti‐zinc transporter 8; CTRLs, control group; Group A, autoimmunity group; IAA, antibodies anti‐insulin; Islet‐AAB, islet autoantibodies; SD, standard deviation.

### 3.1 Subgroup Analysis

In the Group A, only 2 subjects had coeliac disease, 34.3% of participants had a history of autoimmune thyroid disease and 14.9% had APS. Specifically, 8.9% of group A were affected by type 3 APS, 4.5% were affected by type 2 and 1.5% by type 1. Table 5 reports the prevalence of islet‐AAb positivity in Group A according to the presence of specific organ‐related autoantibodies, alone or in combination. Among participants with pituitary autoimmunity (APA), 33.3% were positive for a single islet‐AAb and 9.5% had multiple islet‐AAb positivity. In those with ovarian autoimmunity, 7.1% had a single islet‐AAb and 28.6% had multiple islet‐AAb positivity, a significantly higher prevalence than in participants with other types of organ‐specific autoimmunity (*χ* ^2^ = 17.01; *p* = 0.002). This association appeared even stronger in subjects with combined pituitary and ovarian autoimmunity, 60% of whom showed multiple islet‐AAb positivity (*χ* ^2^ = 23.5; *p* < 0.001). Lower prevalence of islet‐AAb was observed in participants with other organ‐specific endocrine autoimmune conditions, whether single or combined. Clinically, early menopause was reported in 30% of women with anti‐ovarian antibodies and pituitary hormonal alteration in 20% of participants with APA. A history of thyroid autoimmunity was present in 50% of women with anti‐ovarian antibodies and in 23.8% of subjects with APA, but none of these showed multiple islet‐AAb positivity.

**TABLE 5:** Prevalence of islet autoimmunity in subjects with other organ‐specific autoimmunity.

Autoimmunity organ‐specific, *n*	Islet‐AAb+, *n* (%)	One islet‐AAb, *n* (%)	Two islet‐AAb, *n* (%)	Three islet‐AAb, *n* (%)	≥ 2 islet‐AAb, *n* (%)	*p*
APA, 84	36 (42.9)	28 (33.3)	4 (4.8)	4 (4.8)	8 (9.5)	0.002
Ovary, 28	10 (35.7)	2 (7.1)	6 (21.4)	2 (7.1)	8 (28.6)	
AHA, 36	12 (33.3)	12 (33.3)	0 (0)	0 (0)	0 (0.0)	
ACA, 12	4 (33.3)	3 (25)	1 (8.3)	0 (0)	1 (8.3)	
APCA, 30	6 (20.0)	5 (16.7)	1 (3.3)	0 (0)	1 (3.3)	
APA + Ovary, 10	6 (60.0)	0 (0)	4 (40.0)	2 (20.0)	6 (60.0)	< 0.001
APA + ACA,6	2 (33.3)	2 (33.3)	0 (0)	0 (0)	0 (0.0)	
APA+ AHA, 24	6 (25.0)	6 (25.0)	0 (0)	0 (0)	0 (0.0)	
APA + APCA, 12	4 (33.3%)	3 (25.0)	1 (8.3)	0 (0)	1 (8.3)	

*Note: p* value from chi‐square test across single or combined organ‐specific autoimmunity subgroups for prevalence of ≥ 2 islet‐AAb.

Abbreviations: ACA, anti‐adrenal cortex antibodies; AHA, anti‐hypothalamus antibodies; APA, anti‐pituitary antibodies; APCA, anti‐parietal cells antibodies.

When comparing the prevalence of multiple autoantibody (≥ 2 islet‐AAb) positivity between participants with anti‐ovary antibodies and CTRLs, it was significantly higher in the former group (28.6% vs. 5.3%, *p* = 0.003). Similarly, the prevalence was significantly higher among subjects with combined anti‐ovary and anti‐pituitary autoimmunity than in CTRLs (60% vs. 5.3%, *p* < 0.001).

## 4 Discussion

In this retrospective study, adults with autoimmunity against endocrine glands and/or CPGs (Group A) showed a higher prevalence of pancreatic autoimmunity than the control group (CTRLs). Most subjects of Group A had single islet‐AAb positivity and anti‐IA2 was the most frequent subtype, occurring with a frequency significantly higher than in controls. Moreover, mean titres of all tested islet‐AAb, except IAA, were above the 75th percentile of the overall study population in Group A, whereas titres in the control group were generally low or medium‐low. These findings indicate that pancreatic autoimmunity is not uncommon in adults with organ‐specific autoimmunity, even in the absence of known diabetes or a family history of type 1 diabetes.

Subgroup analyses demonstrated that, among participants with organ‐specific autoimmunity, those with ovarian autoimmunity had a markedly higher prevalence of multiple islet‐AAb positivity, particularly when ovarian and pituitary autoimmunity coexisted. Although the first stage of T1D is classically defined by the presence of multiple autoantibodies in paediatric age, autoimmune diabetes in adults often presents with a single islet‐AAb. Anti‐GAD is usually the first autoantibody detected in adults, while anti‐IA2 appear less common as an initial marker of pancreatic autoimmunity [8]. However, anti‐GAD can be found in other autoimmune diseases, whereas anti‐IA2 is more specific for insulitis and its presence is strongly associated with faster progression to clinical T1D [8, 18]. Anti‐IA2 Ab target an intracellular epitope released in case of *β*‐cell damage, so it has been speculated that anti‐IA2 Ab positivity may identify subjects closer to clinical disease [19]. Consistent with this, current ADA guidelines recommend testing anti‐IA2 and/or anti‐ZNT8 when anti‐GAD are negative, but autoimmune diabetes is suspected in adults [6]. Assessment of islet‐AAb other than anti‐GAD has been linked to an approximately threefold higher incidence of slowly progressive autoimmune diabetes in patients not yet treated with insulin therapy [20].

To date, most data on pancreatic autoimmunity in the context of endocrine autoimmune diseases derive from paediatric cohorts with autoimmune thyroid disease or coeliac disease, in whom an increased risk of T1D has been reported [21, 22]. A recent retrospective matched‐cohort study based on real‐world claims data, conducted predominantly in adults, provided findings broadly consistent with those reported in children [23]. Furthermore, a large cohort study investigated incident adult‐onset T1D after adolescent autoimmunity and reported a higher risk of disease, mainly driven by thyroid autoimmunity and coeliac disease; anyway, other endocrine autoimmunity beyond thyroid disease were not included [24]. The present study extends this evidence by exploring islet‐AAb prevalence in less frequently investigated endocrine autoimmune conditions, particularly ovarian and pituitary autoimmunity.

Regarding the relationship between ovarian and pancreatic autoimmunity, previous work in women with T1D has reported an increased risk of premature ovarian failure, likely mediated by mechanisms other than direct ovarian autommunity [25]. More recently, higher frequencies of autoimmune diseases have been described in women with primary ovarian insufficiency [26]. In line with the pattern observed in our cohort, a population‐based study using electronic health records showed that women with POI had a significantly increased risk of multiple autoimmune conditions, including type 1 diabetes and autoimmune hypothyroidism, compared with population rates [26]. Notably, in our study 50% of women with ovarian autoimmunity had a history of autoimmune thyroid disease, but interestingly none of the individuals with ≥ 2 islet‐AAb belonged to this subgroup, suggesting that the association between multiple islet‐AAb positivity and ovarian autoimmunity may be at least partly independent of thyroid autoimmunity.

Pituitary autoimmunity is most commonly reported in association with autoimmune thyroid diseases [14] and HLA‐DQ8, a genotype that also confers susceptibility to T1D, has been found to be frequent in patients with hypophysitis [27]. The high prevalence of multiple islet‐AAb positivity in individuals with combined pituitary and ovarian autoimmunity observed in our study raises the hypothesis that shared genetic or immunological mechanisms might underlie this clustering of autoimmune disorders. Current clinical guidelines of Scientific Societies for autoimmune diseases (excluding primary adrenal insufficiency) do not provide specific recommendations for T1D screening in individuals with pre‐existing autoimmune diseases or a family history of autoimmunity [28]. Our findings support the concept that selected adult subgroups, such as those with ovarian and/or pituitary autoimmunity, may benefit from targeted islet‐AAb screening as part of a personalised risk‐stratification strategy.

Adult‐onset autoimmune diabetes is highly heterogeneous in terms of onset, progression, genetic risk and antibody profile, making a general screening program both challenging and unjustified, despite the rising incidence in recent years. However, given the typically slower rate of beta‐cell loss, adult‐autoimmune diabetes may offer a wide opportunity for intervention with emerging disease‐modifying therapies, such as teplizumab, to prevent progression to clinical stages [29]. In this context identifying adult populations to target screening, such as our cohort, could be clinically very useful.

Our results should be interpreted with caution, as they describe the prevalence of pancreatic autoimmunity in a cohort of adults with other endocrine autoimmune positivity but do not provide definitive insights into the risk of progression to overt type 1 diabetes. Nevertheless, the higher prevalence of multiple islet autoantibody positivity, which is known to be associated with an increased risk of progression, in individuals with ovarian and/or pituitary autoimmunity suggests that this population warrants further investigations. These subgroup findings should be considered exploratory, as they derive from a relatively small sample size. Larger, prospective longitudinal studies are needed to better clarify the association between these pre‐existing endocrine autoimmunity, the risk of developing islet autoimmunity and the progression to overt diabetes.

Moreover, our findings may be most applicable to patients with stable and/or untreated autoimmune diseases, because we excluded subjects with current or recent immunosuppressive therapy in order to avoid bias in autoantibodies detection. Although this approach strengthens internal validity, it reduced the representativeness of the cohort by excluding individuals with more severe or recently treated autoimmune disease.

It is also noteworthy that the prevalence of obesity was higher in group A, although this difference did not reach statistical significance. Obesity and the associated chronic inflammatory state have been implicated in the promotion of autoimmune processes and may contribute to the onset or progression of autoimmune diabetes [30].

## 5 Strengths and Limitations of Study

The present study has limitations that should be acknowledged. First, the lack of detailed glycemic data in participants with pancreatic autoimmunity prevents assessment of the relationship between islet‐AAb positivity and current dysglycaemia or progression to overt diabetes. Second, autoimmune conditions commonly associated with T1D, such as autoimmune thyroid disease and celiac disease, were ascertained only from medical records, which may have led to underreporting or misclassification. Third, the control group cannot be regarded as a truly healthy control group, since participants were evaluated for suspected or confirmed endocrine disorders and autoimmune conditions other than those specifically tested could not be definitively excluded. Accordingly, the control group represents a clinically referred population and the relatively high prevalence of islet autoantibody positivity observed among them should be interpreted in this context.

Nonetheless, this study represents one of the first systematic efforts to characterize pancreatic autoimmunity and multiple islet‐AAb subtypes in adults with relatively rare endocrine autoimmune conditions.

## 6 Conclusion

In summary, adults with autoimmunity against endocrine glands and/or gastric parietal cells have an increased prevalence of pancreatic autoimmunity, with higher titers and more frequent anti‐IA2 positivity than controls. Within this population, ovarian autoimmunity—particularly when combined with pituitary autoimmunity—is associated with a significantly higher prevalence of multiple islet‐AAb positivity. These findings suggest that patients with premature ovarian failure and/or autoimmune pituitary disorders may constitute a high‐risk group for early‐stage T1D. Further large, prospective studies including detailed metabolic assessment and longitudinal follow‐up are needed to confirm these data and to investigate whether these patients could benefit from targeted islet autoantibody screening and closer metabolic monitoring, with the aim of enabling timely intervention and delaying progression to clinical disease.

## Author Contributions

Giuseppe Bellastella conceived the study, drafted it and agreed for all aspects of the work. Michela Di Nuzzo, Paolo Cirillo, Nicole Di Martino, Silvia Angelino and Antonietta Maio collected data, managed data analysis and contributed to the discussion. Miriam Longo, Paola Caruso, Lorenzo Scappaticcio and Maria Ida Maiorino interpreted results and critically reviewed the study. Maria Teresa Vietri performed anti‐islet antibodies assays on participants' samples. Katherine Esposito supervised the project and provided critical insight. All authors reviewed and approved the final version of the manuscript.

## Funding

The authors have nothing to report.

## Conflicts of Interest

The authors declare no conflicts of interest.

## Data Availability

The datasets used and/or analyzed during the current study are available from the corresponding author upon reasonable request.
